# SIRT1 in Liver Diseases: Mechanistic Insights and Therapeutic Prospects

**DOI:** 10.7150/ijbs.117669

**Published:** 2025-11-05

**Authors:** Fucheng Zuo, Junfa Yang, Qixiang Wu, Yaru Yang, Huan Zhou, Yuansong Sun, Tao Xu

**Affiliations:** 1 Inflammation and Immune Mediated Diseases Laboratory of Anhui Province; School of Pharmaceutical Sciences, Anhui Medical University, Hefei, Anhui 230032, China.; 2 Department of Infectious Diseases, Tong Ling People's Hospital, Tongling, Anhui 244000, China.; 3 Department of Emergency Surgery, the Second Affiliated Hospital of Anhui Medical University, Hefei, Anhui 230001, China.; 4 Department of Clinical Pharmacology, the Second Affiliated Hospital of Anhui Medical University, Hefei, Anhui 230001, China.; 5 The First Affiliated Hospital of Anhui Medical University, Clinical Research Hospital, Hefei, Anhui 230000, China.; 6 Key Laboratory of Innovative Pharmaceutical Research and Clinical Evaluation Jointly Constructed by Anhui, Bengbu, Anhui 233000, China.

**Keywords:** SIRT1, liver disease, MASLD, liver fibrosis, HCC

## Abstract

Liver diseases present a formidable global health challenge and rank among the leading causes of morbidity and premature mortality worldwide. Silent information regulator 1 (SIRT1), a nicotinamide adenine dinucleotide (NAD⁺)-dependent histone deacetylase, has emerged as a crucial regulator of various pathophysiological processes, including metabolic homeostasis, inflammatory responses and apoptosis. This evolutionarily conserved enzyme exhibits a disease‑specific expression profile and is subject to tightly regulated mechanisms in diverse liver diseases. In recent years, accumulating evidence has highlighted the critical involvement of SIRT1 dysregulation in the pathogenesis of various liver diseases. In this review, we provide a comprehensive overview of the roles of SIRT1 in multiple liver diseases, including metabolic dysfunction-associated steatotic liver disease (MASLD), alcohol-associated liver disease (ALD), liver fibrosis, and hepatocellular carcinoma (HCC). We further explore the underlying regulatory mechanisms, aiming to establish a rigorous framework to facilitate the clinical translation of SIRT1-targeted therapeutic strategies.

## Introduction

Liver diseases are conditions that disrupt the basic physiological processes and normal functions of the human liver, encompassing both acute and chronic liver diseases. Based on etiology, common liver diseases primarily include infectious liver diseases, metabolic liver diseases (e.g., metabolic dysfunction-associated steatotic liver disease (MASLD) and alcohol-associated liver disease (ALD), and immune-related liver diseases^1^, ^2^. A 2025 epidemiological study revealed that over 1.72 billion people worldwide suffer from liver diseases, resulting in more than 2 million deaths each year, with disability-adjusted life years (DALYs) attributable to ALD and MASLD on an upward trend^3^. Although ongoing advancements in clinical research have brought about improved clinical outcomes for a broader range of liver diseases, as evidenced by the fact that direct-acting antiviral agents (DAAs) have successfully eradicated the virus in over 98% of hepatitis C virus (HCV) patients, effective therapeutic strategies for conditions such as ALD and hepatocellular carcinoma (HCC) remain limited^3^-^5^. In recent years, promising biomarkers and therapeutic targets have garnered increasing attention, with the aim of enhancing early diagnostic capabilities and paving the way for novel therapeutic approaches.

Silent information regulator 1 (SIRT1), also known as Sirtuin 1, is an NAD⁺-dependent deacetylase that regulates a variety of biological processes by deacetylating multiple proteins, including both histones and non-histone proteins^6^, ^7^. Due to its deacetylase activity, SIRT1 plays a crucial role in regulating cellular metabolism, oxidative stress, aging, inflammation, and genomic stability. Numerous studies have evidenced its connection with multiple clinical disorders, including neurodegenerative diseases (e.g., Alzheimer's disease and Parkinson's disease)^8^, ^9^, inflammatory demyelination^10^, osteoarthritis^11^, and metabolic diseases^12^. Notably, in the liver, SIRT1 regulates genes involved in both lipogenesis and lipid catabolism, thereby preventing excessive lipid accumulation^13^. It can also upregulate the expression of antioxidant genes, such as heme oxygenase-1 (HO-1) and NAD(P)H quinone dehydrogenase 1 (NQO1), thereby alleviating liver oxidative stress. Consequently, the investigation of SIRT1 in liver pathophysiology has gradually emerged as a focal point of research.

Even though SIRT1 has undergone comprehensive research in aging and metabolic modulation, its specific functions and regulatory mechanisms in various liver disease settings remain incompletely understood. This review provided a comprehensive summary of the pivotal role of SIRT1 in various liver diseases, elucidates its molecular regulatory mechanisms, and highlights the application of advanced technologies in related research, ultimately aiming to link these insights to potential therapeutic opportunities.

## Overview of SIRT1

**Figure 1:**
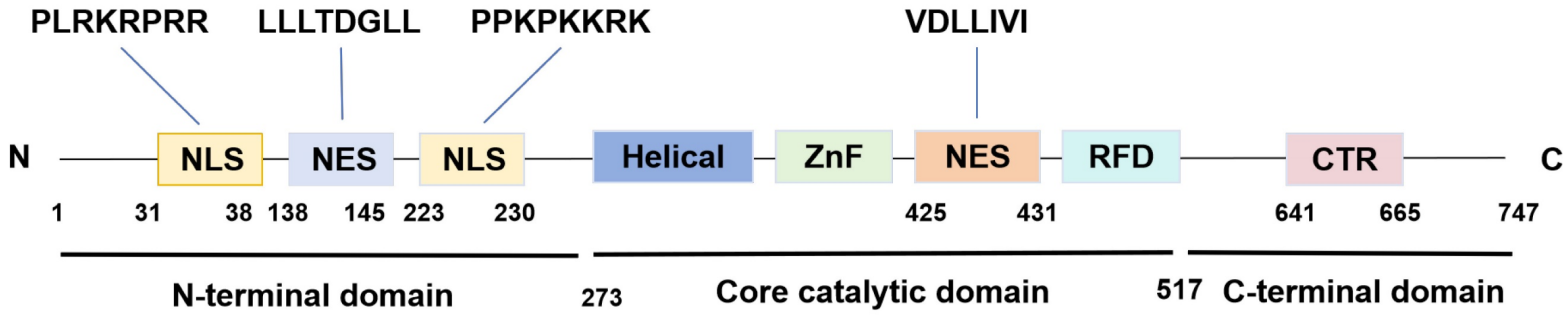
** The structure of SIRT1.** SIRT1 protein consists of 747 amino acids (aa) and has a highly conserved core catalytic domain (aa 273-517) containing a Rossmann fold domain (RFD),zinc-binding domain (ZnF) and a helical module, SIRT1 encompasses 2 nuclear localization signals (NLS, aa 31-38 and 223-230) and 2 nuclear exportation signals (NES, aa 138-145 and 425-431), which is essential for the nucleocytoplasmic shuttling of SIRT1. C-terminal regulatory domain (CTR, aa 641-665) is critical to regulate SIRT1 activity.

SIRT1 is the first discovered evolutionarily conserved member of the mammalian sirtuin family (SIRT1-SIRT7), initially discovered in humans by Frye in 1999^14^. It is predominantly localized in the nucleus while also capable of shuttling between the nucleus and the cytoplasm^15^. The human SIRT1 gene is located at chromosome 10q21.3 and encodes a protein consisting of 747 amino acids. This protein architecture features a C-terminal elongation, an N-terminal segment containing two functional nuclear localization signals (NLSs) and two nuclear export signals (NESs), along with a central catalytic domain^16^. The catalytic core, composed of 277 residues, is partitioned into two distinct subdomains. The larger subdomain features a Rossmann fold structure, which facilitates NAD⁺ binding. Conversely, the smaller subdomain consists of two insertions within the NAD^+^-binding region, comprising a helical module (residues 269-324) and a Zn^2+^-binding module (residues 362-419)^17^. Within the cleft between the two subdomains, the substrate's acetylated residue binds to NAD^+^, initiating the catalytic reaction. During this process, NAD⁺ undergoes hydrolysis, facilitating the transfer of the acetyl moiety to the 2'-hydroxyl group of ADP-ribose. This biochemical process culminates in the generation of nicotinamide and 2'-O-acetyl-ADP-ribose as the end products^18^. Furthermore, the NES and NLS are essential for nucleocytoplasmic shuttling of SIRT1, with its translocation between the nucleus and cytoplasm that governs both its subcellular distribution and its action on histone and non-histone targets **(Figure 1)**^19^.

SIRT1 is present in multiple human tissues and cells, such as skeletal muscle cells, hepatocytes, pancreatic β-cells, white adipocytes, neurons, and cardiomyocytes, where it exerts dynamic transcriptional regulatory functions^20^-^24^. The expression levels of SIRT1 are modulated by a broad range of transcription factors. For instance, transcription factors including p53 and hypermethylated in cancer 1 (HIC1) have been shown to play a role in downregulating SIRT1 transcription, whereas E2F transcription factor 1 (E2F1) and c-Myc can induce its expression by binding to specific sites within the SIRT1 promoter^25^-^27^. Additionally, SIRT1 is subject to post-transcriptional regulation by various non-coding RNAs (ncRNAs), particularly microRNAs (miRNAs). Several miRNAs, such as miR-9, miR-22, miR-34a, miR-143, miR-146 and miR-181a, interact with the 3' untranslated region (3'UTR) of SIRT1 mRNA, thereby repressing its translation or promoting mRNA degradation^28^. Certain miRNAs such as miR-34a can also target nicotinamide phosphoribosyl transferase (NAMPT), thereby reducing SIRT1 activity^29^. Notably, SIRT1 plays a pivotal role in maintaining normal liver physiology. Specifically, SIRT1 can enhance the activity of key antioxidant enzymes, including superoxide dismutase (SOD), catalase (CAT), and glutathione peroxidase (GPX) by deacetylating forkhead box O (FOXO) transcription factors and peroxisome proliferator-activated receptor γ coactivator 1α (PGC-1α), thereby alleviating oxidative stress^6^. Besides, SIRT1 is essential for the regulation of lipid and glucose metabolism, contributing to the maintenance of normal liver metabolic function^13^. Consequently, SIRT1 plays a critical role in maintaining physiological homeostasis. As research advances, SIRT1 has garnered widespread attention in the study of various liver diseases.

## Functional role of SIRT1 in liver diseases

**Figure 2:**
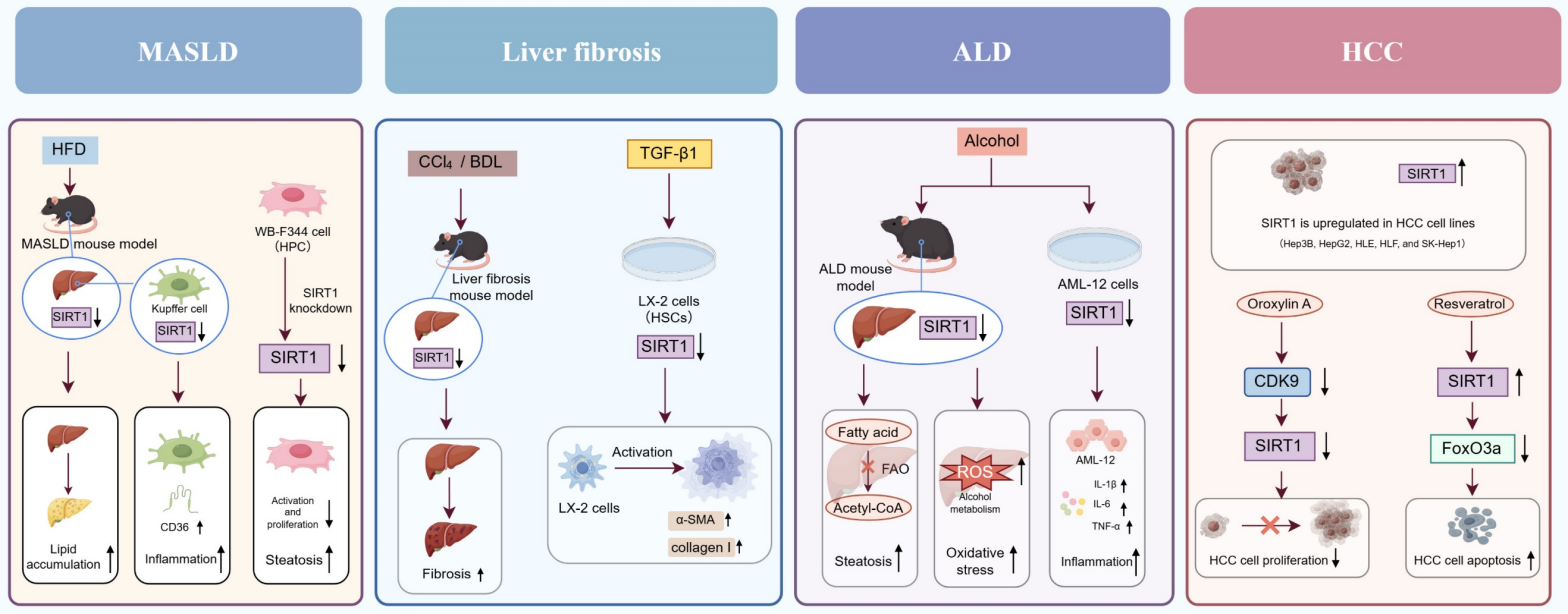
**The function role of SIRT1 in various liver diseases.** In MASLD, SIRT1 downregulation exacerbates hepatic lipid accumulation, inflammation, and steatosis. In liver fibrosis, reduced SIRT1 expression activates HSCs and promotes the secretion of α-SMA and collagen I. In ALD, SIRT1 downregulation aggravates hepatic steatosis, oxidative stress, and inflammation. In HCC, SIRT1 exhibits a dual role by promoting both HCC cell proliferation and apoptosis.

In recent studies, accumulating data have demonstrated that SIRT1 is crucial for preserving normal liver function and influencing liver disease progression. This section provides a systematic overview of the functional roles of SIRT1 in various common liver diseases (**Figure 2**).

### Metabolic dysfunction-associated steatotic liver disease

MASLD is a new term proposed in 2023 by an international expert consensus to replace non-alcoholic fatty liver disease (NAFLD), with the aim of more accurately reflecting the causal relationship between the condition and metabolic dysregulation^30^. As the primary cause of chronic liver disease worldwide, MASLD has become a significant public health issue. Recent epidemiological investigations revealed that MASLD impacts approximately 38% of the adult population and 7 - 14% of children and adolescents^31^, ^32^. By 2040, the prevalence of this disease among adults is projected to exceed 55%, highlighting the need for urgent preventive and therapeutic strategies^33^. This condition is invariably accompanied by at least one metabolic risk factor, such as obesity, type 2 diabetes, hypertension, dyslipidemia, or insulin resistance^34^. Its pathogenesis involves dysregulation of lipid metabolism, ectopic lipid deposition secondary to insulin resistance, mitochondrial dysfunction, and chronic inflammatory responses^35^.

Emerging evidence indicated that SIRT1 expression plays a critical role in the progression of MASLD, with its suppression correlating with the severity of liver injury^36^. For example, Li et al. found that SIRT1 knockdown led to significant increases in body weight and liver weight in high-fat diet (HFD)-induced MASLD mice, exacerbated liver lipid deposition and steatosis, and induced substantial inflammatory cell infiltration^37^. Recently, Liu et al. have shown that SIRT1 deacetylated and activated PGC-1α, thereby enhancing fatty acid β-oxidation (FAO) while suppressing the transcription of key lipogenic genes, such as fatty acid synthase (FAS) and acetyl coenzyme A carboxylase (ACC). This coordinated regulation effectively inhibited *de novo* lipogenesis and liver lipid accumulation^38^. Additionally, several studies have confirmed that increasing SIRT1 expression and activity can alleviate lipid accumulation and oxidative stress in HFD-induced mouse model of MASLD^39^, ^40^. Notably, during the progression of MASLD, SIRT1 exhibits cell-specific activity, exerting distinct effects across different liver cell types. In 2018, Yao et al. demonstrated that in a palmitic acid (PA)-induced AML-12 murine parenchymal hepatocyte model of MASLD, SIRT1 expression is significantly downregulated, thereby impairing downstream lipid metabolic regulators such as sterol regulatory element-binding protein 1 (SREBP-1)^41^. This impairment disrupts FAO and leads to excessive lipid accumulation. For Kupffer cells, the liver-resident macrophages, SIRT1 expression is also downregulated during the progression of MASLD. In 2018, Niu et al. found that HFD treatment significantly downregulated both mRNA and protein levels of SIRT1 in Kupffer cells of mice, while upregulating CD36 expression and increasing the secretion of inflammatory cytokines, thereby exacerbating hepatic inflammatory cell infiltration. Correspondingly, SIRT1 activation can downregulate cluster of differentiation 36 (CD36) expression in Kupffer cells, thereby inhibiting their activation and alleviating liver inflammation^42^. Moreover, in 2021, Li et al. have shown that SIRT1 deficiency impeded the activation and proliferation of WB-F344 cells (rat hepatic progenitor cells, HPCs), thereby hindering the HPCs-mediated restoration from liver steatotic injury^37^. Accordingly, pharmacological modulators of SIRT1 represent promising therapeutic candidates for MASLD. As an illustration, SRT1720, a compound known to activate SIRT1, has been extensively validated for its effect in alleviating liver steatosis associated with MASLD. This beneficial effect is evidenced by the reduction in serum alanine aminotransferase (ALT) and aspartate aminotransferase (AST) concentrations, along with a decrease in the expression of liver inflammatory cytokines including tumor necrosis factor-α (TNF-α) and monocyte chemoattractant protein-1 (MCP-1)^43^, ^44^.

In recent years, the interplay between gut microbiota and host metabolism has emerged as a research hotspot. Emerging evidence has demonstrated that gut microbiota and its derived metabolites (e.g., bile acids and short chain fatty acids, SCFAs) affect the progression of liver injury by modulating SIRT1^45^. SCFAs are carboxylic acids with aliphatic tails of 1-6 carbon atoms and are major products of gut microbes^46^. Several studies have reported that changes in the levels of SCFAs are closely related to the pathogenesis of MASLD. For example, SIRT1 can be directly or indirectly activated by SCFAs, which activates cellular antioxidant mechanisms and downregulates the expression of pro-inflammatory cytokines, attenuating liver injury^47^. In addition, as common metabolic products of gut microbiota, bile acids exert effects during the early stages of various types of liver diseases including MASLD^48^, ^49^. For instance, glycochenodeoxycholic acid (GCDCA), a toxic bile acid, has been shown to directly reduce the expression and activity of SIRT1 protein, thereby impairing the deacetylation of PGC-1α and leading to disrupted liver lipid metabolism^50^. Notably, in 2023, Yang et al. have indicated that didymin, a flavonoid extracted from citrus peels, mitigates MASLD via the activation of SIRT1. In a HFD-induced mouse model of MASLD and Palmitic acid (PA)-treated AML-12 cells, didymin increased SIRT1 expression and directly activated SIRT1, thereby enhancing mitochondrial biogenesis and function, reducing apoptosis, and promoting lipophagy, ultimately alleviating MASLD^51^. Furthermore, Tian et al. demonstrated that the Xie Zhuo Tiao Zhi (XZTZ) formula alleviated MASLD by modulating SIRT1, which suppressed NLRP3 inflammasome-mediated pyroptosis in macrophages, reduced M1 polarization while promoting M2 polarization, and concurrently improved mitochondrial function and lipid metabolism^52^. As a complement to the findings on natural products and herbal formulas, recent studies have also shown that S. boulardii (a gut probiotic) can increase SIRT1 expression as well as reduce lipid accumulation and oxidative stress in fructose-induced MASLD rats^53^. In summary, SIRT1 expression exerts a pivotal protective effect in the progression of MASLD, underscoring the critical importance of developing SIRT1-activating agents for its prevention and treatment.

### Liver fibrosis

Liver fibrosis is a pathological repair response triggered by chronic liver injury^54^. This condition can be triggered by various etiologies, including viral hepatitis, alcohol consumption and liver steatosis^55^. It is defined by the abnormal accumulation of extracellular matrix (ECM) components, particularly crosslinked collagens type I and type III (COL1A1 and COL3A1)^56^. This pathological process results in the distortion of the normal liver architecture and subsequent impairment of liver function^56^. In 2024, an investigation performed by Kim et al. incorporated 45 studies with a cumulative sample size of 56,969 individuals, revealing a 7.3% prevalence of clinically significant liver fibrosis among the general population^57^. Generally, the central mechanism of liver fibrosis involves the activation of hepatic stellate cells (HSCs). Upon stimulation by liver injury signals, particularly transforming growth factor-β (TGF-β), HSCs transform into myofibroblasts, secreting large amounts of fibrosis-associated proteins, ultimately leading to excessive extracellular matrix (ECM) deposition^58^.

Emerging studies have identified SIRT1 as a critical regulator in the fibrotic processes of multiple organs, including the heart^59^, kidney^60^, lung^61^, and liver^62^. Notably, in both carbon tetrachloride (CCl₄)- and bile duct ligation (BDL)-induced mouse models of liver fibrosis, a marked downregulation of SIRT1 expression has been observed. Mechanistic investigations suggested that inhibition of SIRT1 activity exacerbates fibrogenesis by promoting HSCs activation, with upregulated expression of α-smooth muscle actin (α-SMA) and collagen I. Conversely, SIRT1 activation suppresses fibrotic markers, including α-SMA and collagen I, demonstrating significant antifibrotic effects^63^. In 2015, Wu et al. demonstrated that in a CCl_4_ induced mouse model of liver fibrosis, the expression of SIRT1 in liver tissues was significantly decreased, whereas in a spontaneous fibrosis regression model, SIRT1 expression was restored to normal levels. Subsequently, in an *in vitro* model of liver fibrosis established by treating LX-2 cells (human HSCs) with TGF-β1, SIRT1 expression was markedly downregulated. Overexpression of SIRT1 in LX-2 cells via transfection with the pECE-SIRT1 plasmid significantly suppressed the expression of α-SMA and COL1A1. These findings indicated that SIRT1 downregulation was particularly pronounced in HSCs during liver fibrosis, and restoring its activity effectively inhibited aberrant HSCs activation. Furthermore, the long ncRNA MALAT1 (lncRNA MALAT1) was significantly upregulated in TGF-β1-induced LX-2 cells and exhibited a negative correlation with SIRT1 expression level. Inhibition of lncRNA MALAT1 via RNA interference restored SIRT1 levels and markedly reduced the expression of fibrotic markers, including α-SMA and COL1A1. Therefore, lncRNA MALAT1 may exacerbate the fibrotic process by suppressing the expression of SIRT1^64^. Of note, during the progression from steatotic liver disease to fibrosis, SIRT1 downregulation in parenchymal hepatocytes and Kupffer cells leads to lipid metabolic disturbances that facilitate HSCs activation. TGF-β derived from activated HSCs further suppresses SIRT1 expression in hepatocytes, forming a positive feedback loop that perpetuates lipid accumulation, inflammatory responses, and ECM deposition. Additionally, in 2021, Luo et al. found that SIRT1 expression was downregulated in hepatic sinusoidal endothelial cells (HSECs) during CCl_4_-induced liver fibrosis, leading to its defenestration and capillarization of hepatic sinusoids. Whereas overexpressing SIRT1 in HSECs deacetylated p53, thereby suppressing oxidative stress-induced senescence of HSECs, attenuating CCl_4_-induced defenestration and the progression of liver fibrosis^65^. Moreover, in 2024, Rungratanawanich et al. demonstrated that gut-specific SIRT1 knockout mice exhibited exacerbated liver fibrosis in thioacetamide (TAA)-induced gut leakiness and liver fibrosis dual model. Further melatonin pretreatment restored SIRT1 expression and reduced protein acetylation in both the gut and liver of model mice, ultimately ameliorating TAA-induced gut leakiness and liver fibrosis^66^. Based on these results, melatonin emerges as a potentially viable and secure dietary supplement option for individuals suffering from liver fibrosis. Overall, SIRT1 plays a critical protective role against liver fibrosis, and modulating SIRT1 represents a promising therapeutic strategy for liver fibrosis.

### Alcohol-associated liver disease

ALD is a progressive liver disorder caused by prolonged excessive alcohol consumption. Its presentation varies from asymptomatic liver steatosis to fibrosis, cirrhosis, alcohol-related hepatitis, and related sequelae^67^. In 2023, a meta-analysis involving 513,278 individuals revealed that the prevalence of ALD was 3.5% in the general population and 26.0% among high-risk drinkers^68^. Currently, no effective targeted therapy exists for ALD, and strict alcohol abstinence remains the most effective measure for improving patient prognosis^69^, ^70^. Consequently, it is imperative to discover pertinent therapeutic targets and create corresponding drugs for ALD.

The pathogenesis of ALD crucially involves the metabolism of alcohol in the liver, primarily by alcohol dehydrogenase (ADH) and the microsomal ethanol-oxidizing system (MEOS), which generates substantial reactive oxygen species (ROS). This triggers oxidative stress, leading to hepatocyte damage^71^. SIRT1 has been shown to exert liver protective effects in Lieber-DeCarli alcohol diet induced ALD mice, through mitigating ROS-induced oxidative stress. Specifically, chronic alcohol consumption leads to a downregulation of SIRT1 expression in liver, while preservation of SIRT1 expression and activity results in the upregulation of antioxidant-related genes (e.g., GPX2 and SOD1), reduction of intrahepatic ROS levels, and attenuation of lipid peroxidation-induced damage. Conversely, silencing of SIRT1 markedly abolishes these protective effects against oxidative stress, confirming that SIRT1 is a pivotal regulator of redox homeostasis in ALD^72^. In addition, Liu et al. innovatively applied positron emission tomography/computed tomography (PET/CT) imaging to visualize and quantitatively assess hepatic SIRT1 expression in early-stage ALD mice, revealing a significant downregulation, which was further confirmed by Western blot analysis^73^. In 2012, Yin et al. found that in an *in vitro* AML-12 cell model of ALD, alcohol induced miR-217 expression dose-dependently. miR-217 can directly bind to the 3'-UTR of SIRT1 mRNA, thereby suppressing SIRT1 expression and leading to abnormal lipid accumulation in parenchymal hepatocyte^74^. Subsequently, in 2015, Yin et al. revealed that during the progression of ALD, alcohol treatment markedly enhanced lipopolysaccharide (LPS)-induced upregulation of miR-217 in Kupffer cells. Then miR-217 suppressed SIRT1 expression, thereby promoting the production of pro-inflammatory cytokines in Kupffer cells and exacerbating hepatic inflammatory injury^75^. Of note, in 2024, Liu et al. further revealed that alcohol exposure markedly upregulated bromodomain-containing protein 4 (BRD4) expression in AML-12 cells. BRD4 can directly bind to the promoter region of SIRT1, repressing its transcription and thereby impairing autophagic activity and lipid metabolic function, ultimately exacerbating hepatic injury^76^.

Given its pivotal role in ALD, SIRT1 has been established as a central orchestrator of liver protection in ALD, operating through interconnected metabolic and immunomodulatory mechanisms. As an NAD^+^-dependent deacetylase, SIRT1 exerts its therapeutic effects by coordinately regulating lipid homeostasis through dual modulation of FAO activation and lipogenesis suppression, effectively counteracting alcohol-induced liver steatosis^77^. Crucially, in addition to regulating lipid metabolism, SIRT1 also alleviates lipid peroxidation induced by ROS generated during alcohol metabolism. Therefore, SIRT1 plays a crucial role in mitigating metabolic liver injury during the progression of ALD. Moreover, SIRT1 reduces the production of inflammatory cytokines, including TNF-α, interleukin-6 (IL-6), and interleukin-1β (IL-1β). Through this mechanism, SIRT1 effectively dampens the inflammatory cascade in the liver, which is particularly relevant in the context of ALD. For example, salvianolic acid A (SalA) has been shown to alleviate ALD in both ALD mouse models and AML-12 cells through upregulating SIRT1, promoting autophagosome-lysosome fusion, activating autophagy, as well as reducing inflammation and lipid accumulation^78^. In 2024, Jiang et al. indicated that Saikogenin A (SGA), a metabolite of Saikosaponin A (SSa) from Radix Bupleuri, improves alcohol-induced liver injury by targeting SIRT1 to modulate lipid metabolism in both the National Institute on Alcohol Abuse and Alcoholism (NIAAA)-based mouse model and HepG2 cells (human HCC cells) model of ALD^79^. Furthermore, a recent study by Ma et al. employing single-cell sequencing demonstrated that miR-155-5p, which is highly expressed in Kupffer cells of ALD patients, can target and inhibit the transcription of SIRT1. And dihydromyricetin (DMY) has been shown to downregulate miR-155-5p, thereby restoring the normal expression of SIRT1, alleviating hepatocyte senescence and inflammatory injury^80^. Thus, SIRT1 exerts protective effects in ALD by alleviating liver injury through multiple mechanisms. This provides a potential therapeutic target and conceptual framework for developing novel treatment strategies for ALD, with promising implications for advancing its management.

### Hepatocellular carcinoma

HCC represents the predominant subtype of primary liver cancer. Epidemiological data indicated that it constitutes nearly 90% of all primary liver cancer incidences^81^. In 2022, HCC was globally recognized as the sixth most commonly diagnosed cancer and the third leading cause of cancer-related death^82^. Its development is closely associated with chronic liver diseases such as viral hepatitis, ALD, MASLD, and liver cirrhosis. These chronic liver injuries lead to the accumulation of deoxyribonucleic acid (DNA) mutations, aberrant proliferation, and epigenetic alterations in hepatocytes, while concurrent microenvironmental disturbances (e.g., inflammatory cytokine dysregulation and aberrant angiogenesis) collectively drive the process of carcinogenesis^83^. Unfortunately, due to its late-stage diagnosis in most cases, the prognosis for HCC is dismal, with a five-year survival rate below 20%^81^.

**Figure 3:**
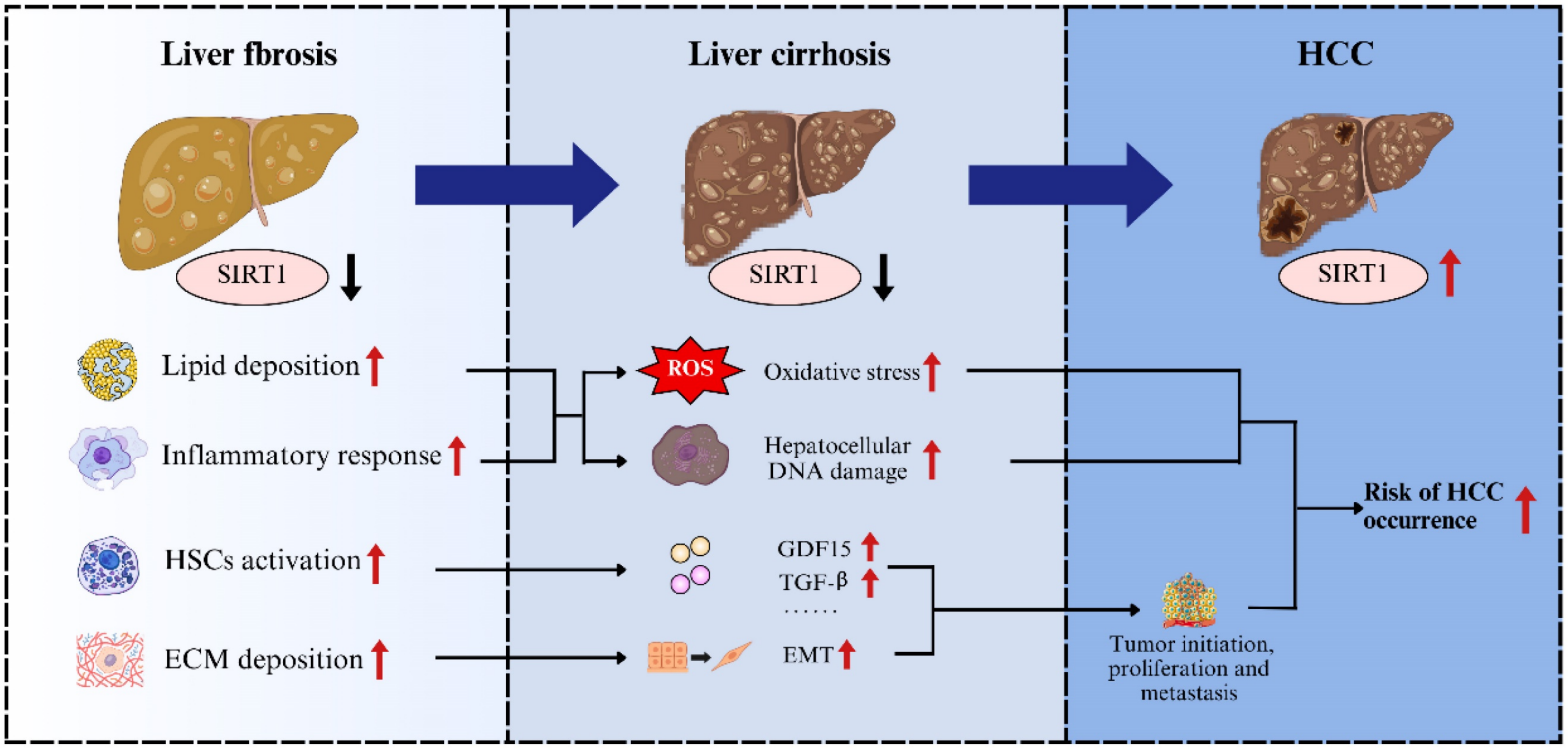
**The contribution of SIRT1 to the transition between liver fibrosis and HCC.** During liver fibrosis, downregulation of SIRT1 leads to lipid accumulation, exacerbated inflammatory responses, actived HSCs and increased extracellular matrix deposition, thereby promoting the progression from fibrosis to cirrhosis and elevating the risk of HCC.

Previous studies have indicated that SIRT1 exhibits a dual role in cancer biology, acting as either a tumor suppressor or a tumor promoter, depending on the specific tumor-specific carcinogenic pathways^84^. For example, in malignant tumors such as colorectal cancer, pancreatic cancer, prostate cancer, and skin cancer, SIRT1 primarily exerts tumor-suppressing effects. Whereas in breast cancer, lung cancer, cervical cancer, endometrial cancer, and ovarian cancer, its effects tend to promote tumor growth^85^-^89^. This dual functionality highlighted the complexity and environment-dependence of SIRT1's involvement in tumorigenesis. In general, advanced liver fibrosis progressed to cirrhosis, which carried a substantial risk of further developing into HCC (**Figure 3**). During the progression of liver fibrosis, SIRT1 expression is downregulated, which concurrently disrupts lipid metabolism, exacerbates inflammation, and promotes ECM deposition. Disruption of lipid metabolism leads to steatosis and lipotoxicity, accompanied by enhanced inflammatory responses that exacerbate oxidative stress and hepatocellular DNA damage, particularly within the cycle of repeated hepatocyte death and compensatory regeneration^90^. Meanwhile, ECM deposition increases liver tissue stiffness, which can be sensed and transduced, thereby promoting hepatocytes proliferation, survival, and epithelial-to-mesenchymal transition (EMT), a process that is critical for tumor initiation, metastasis, and therapeutic resistance^91^. Moreover, downregulation of SIRT1 during liver fibrosis activates HSCs, which secrete growth differentiation factor 15 (GDF15), TGF-β, and other cytokines that not only promote fibrogenesis but also support the growth and proliferation of HCC cells^92^, ^93^. Taken together, the downregulation of SIRT1 during liver fibrosis could induce a series of cellular impairments and alterations in liver microenvironment, which substantially contribute to the progression toward cirrhosis and markedly increase the risk of HCC development. However, unlike in the stages of fibrosis or cirrhosis, during the development of HCC, SIRT1 often exhibits pro-oncogenic properties and is associated with poor prognosis. For instance, Portmann et al. demonstrated that SIRT1 expression is significantly elevated in HCC tissues compared to normal liver tissue or adjacent non-tumorous tissue from the human HCC patients. Consistent with these findings, elevated SIRT1 expression has also been observed in various human HCC cell lines *in vitro*, including Hep3B, HepG2, HLE, HLF, and SK-Hep1^94^. Given the significant elevation of SIRT1 in HCC, further investigations into its functional role have been conducted. Researchers discovered that knockdown of SIRT1 using shRNAs (shSIRT1_3206 and shSIRT1_1958) or pharmacological inhibition with cambinol (SIRT1 inhibitor) in HepG2 cells induced marked morphological alterations, decreased proliferative capacity, and led to G1 phase cell cycle arrest^94^. Conversely, overexpression of SIRT1 was shown to promote the proliferation of Hep3B cells^95^. Furthermore, in orthotopic xenograft mouse model of HCC, SIRT1 knockdown reduced tumor incidence, and cambinol treatment significantly inhibited tumor growth of HCC^94^. These results indicated that SIRT1 plays an important pro-tumorigenic role in the progression of HCC.

Of note, the cyclin-dependent kinase (CDK) family comprises a group of serine/threonine protein kinases that regulate cell cycle progression through phosphorylation of specific target proteins. Yao et al. demonstrated that CDK9 phosphorylated SIRT1 at Ser47 to enhance its deacetylase activity. Subsequently, SIRT1 deacetylated p53 at Lys382, which reduced the stability of p53, leading to loss of tumor-suppressive function, thereby promoting HCC cell proliferation and survival. Their study further revealed that Oroxylin A (CDK9 inhibitor) suppressed SIRT1 phosphorylation, reduced p53 deacetylation and restored its acetylation and activity, ultimately inhibiting HCC progression. These findings highlighted that targeting the CDK9-SIRT1 axis reverses SIRT1-driven destabilization of p53 and provides a therapeutic strategy against HCC^96^. Interestingly, Chai et al. found that SIRT1 activation appears to exert an inhibitory effect on HCC. Their study demonstrated that resveratrol, a polyphenolic compound, enhances SIRT1 expression and activity, thereby suppressing the FOXO3a phosphorylation, promoting apoptosis in HCC cell lines^97^. To summarize, the dual modulatory role of SIRT1 in HCC underscores its importance as a pivotal research target, and a comprehensive elucidation of its mechanisms is essential for guiding the design of HCC therapies in the future.

## Regulatory mechanism of SIRT1 in liver disease

**Figure 4:**
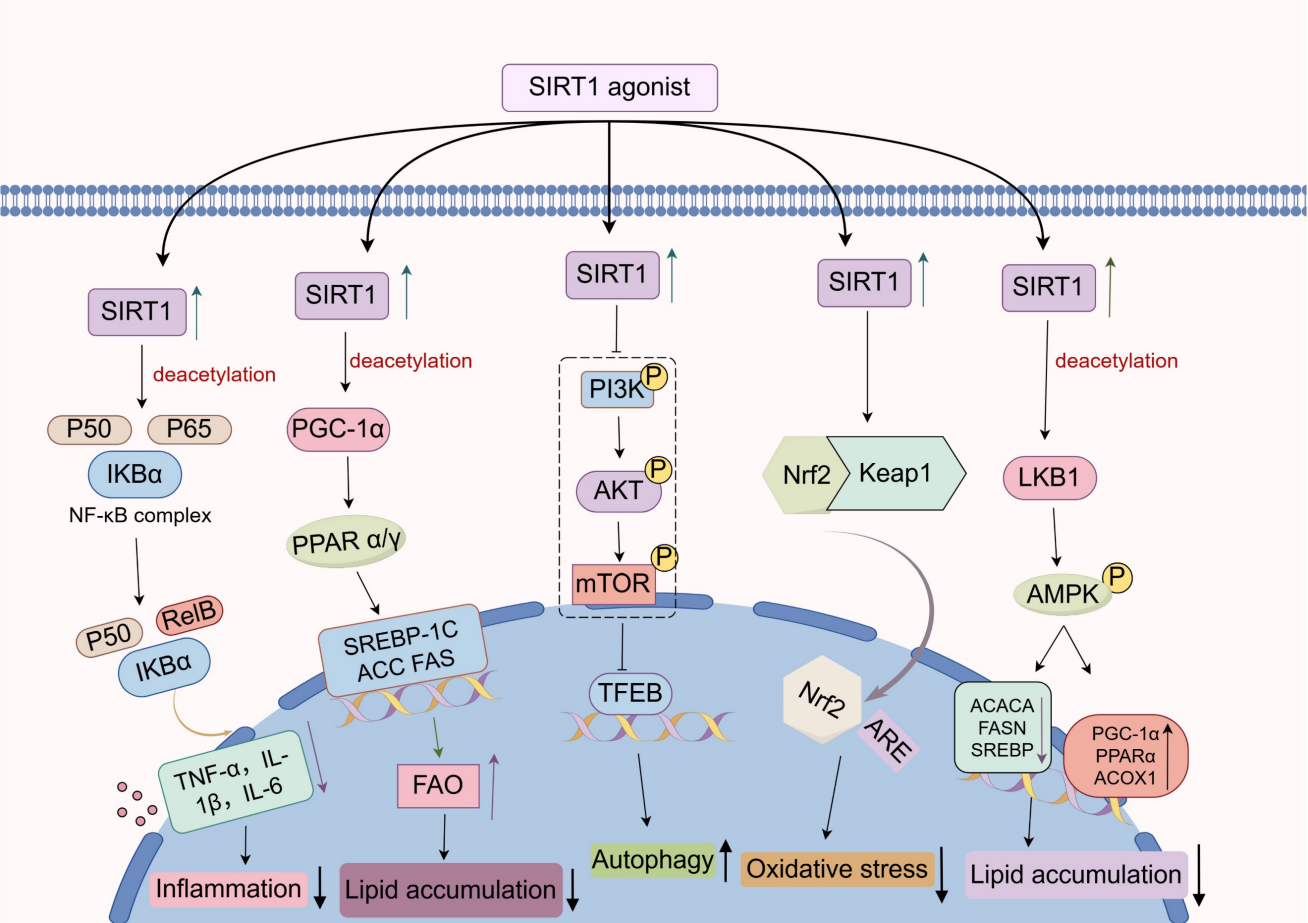
** Regulatory mechanism of SIRT1 in liver disease.** As an NAD⁺-dependent deacetylase, SIRT1 can deacetylate key components within several signaling pathways, including NF-κB, PPAR, PI3K/Akt, Nrf2, and AMPK, thereby modulating the expression of downstream target genes and influencing the progression of liver diseases.

With the continual advances in our understanding of pathophysiology, the pivotal mechanisms by which SIRT1 modulates liver disease progression have become increasingly evident. In this section, we review current studies on the signaling pathways involved in SIRT1-mediated regulation of liver pathology. (**Figure 4**).

### NF-κB signaling pathway

The nuclear factor κB (NF-κB) signaling pathway is a pivotal regulator of inflammation, immune responses, cell proliferation, and apoptosis^98^, ^99^. Its family members include RelA (p65), RelB, c-Rel, and p50^100^. Generally, the NF-κB signaling pathway can be activated through two distinct mechanisms. The canonical NF-κB signaling pathway is triggered by either pro-inflammatory cytokines or pathogen-associated molecular patterns (PAMPs). Upon activation, NF-κB is released from its inhibitory complex and translocates into the nucleus, where it functions as a transcriptional regulator to control the expression of a wide array of target genes. These genes include those encoding pro-inflammatory cytokines such as IL-6 and TNF-α, chemokines, and anti-apoptotic proteins like B-cell lymphoma 2 (Bcl-2)^101^. In contrast, the non-canonical pathway is triggered by ligands such as CD40 and B-cell activating factor (BAFF), relying on NF-κB - inducing kinase (NIK) and inhibitor of κB kinase α (IKKα)-mediated conversion of p100 to p52. The p52 then forms dimers with RelB to control lymphoid organogenesis and adaptive immunity^102^.

Notably, He et al. demonstrated that overexpression of SIRT1 in a CCl_4_-induced rat model of liver fibrosis led to reduced levels of NF-κB p65 in liver, decreased plasma LPS concentrations, and attenuated liver fibrosis. Further *in vitro* experiments showed that LPS-induced RAW 264.7 cells (mouse macrophages) had reduced SIRT1 expression, increased NF-κB acetylation, elevated secretion of pro-inflammatory cytokines, and differentiated into M1 pro-inflammatory type^103^. In contrast, activation of SIRT1 significantly suppressed the activation of LPS-induced RAW 264.7 cells. Mechanistically, LPS stimulation promotes the acetylation level of NF-κB, thereby activating the downstream NOD-like receptor protein 3 (NLRP3) inflammasome, which promotes caspase-1 activation and the subsequent release of pro-inflammatory cytokines such as IL-1β and TNF-α. SIRT1 acts as an NAD⁺-dependent deacetylase that can directly interact with the p65 subunit of the NF-κB complex and deacetylate it at lysine 310, subsequently repressing NF-κB transcriptional activity^103^. Consequently, activation of SIRT1 can attenuate the inflammatory response during liver fibrogenesis through inhibiting the NF-κB signaling pathway. Similarly, in 2023, Yan et al. found that SIRT1 activation promoted NF-κB deacetylation, thereby suppressing the transcription of pro-inflammatory cytokines in PA-induced HepG2 cells. In parallel, the inhibition of NF-κB led to the downregulation of key lipogenic proteins, including SREBP-1, fatty acid synthase (FAS), and the fatty acid transporter CD36, resulting in reduced liver triglyceride (TG) and total cholesterol (TC) accumulation. This was accompanied by restored phosphorylation of insulin receptor substrate 2 (IRS-2) and improved insulin resistance^104^. Moreover, in a methionine and choline deficient (MCD) diet-induced mouse model of non-alcoholic steatohepatitis (NASH), activation of SIRT1 inhibited NF-κB signaling pathway and exerted anti-steatotic, anti-inflammatory, and anti-apoptotic effects^105^. Of interest, Rhodiola rosea glycosides, a class of glycosidic compounds extracted from Rhodiola rosea, markedly alleviated LPS-induced liver inflammation and injury through this signaling pathway, while ginsenosides, triterpenoid saponins extracted from plants of the genus Panax, attenuated alpha-naphthylisothiocyanate (ANIT)-triggered cholestatic liver damage via analogous mechanisms^106^, ^107^. Furthermore, Cyanidin-3-O-β-glucoside (Cy-3-G), a dietary anthocyanin abundantly present in fresh produce, has been shown to reduce liver inflammation and injury by directly interacting with and deacetylating the NF-κB p65 subunit via activating SIRT1^108^. In conclusion, the NF-κB signaling pathway plays a crucial regulatory role in the progression of liver diseases, and a deeper understanding of SIRT1's involvement in the NF-κB signaling pathway offers great potential for developing novel strategies and therapies for liver diseases.

### PPAR signaling pathway

The peroxisome proliferator-activated receptor (PPAR) signaling pathway comprises a class of ligand-activated nuclear transcription factors that play a central role in the regulation of energy metabolic homeostasis^109^. This signaling pathway includes three major isoforms: PPARα, PPARγ, and PPARδ/β. They exert function by forming heterodimers with retinoid X receptors (RXRs), which specifically bind to peroxisome proliferator response elements (PPREs) within the promoter regions of target genes, thereby regulating gene transcription^110^.

Among the PPARs, PPARα is predominantly expressed in the liver, where it governs fatty acid oxidation and ketogenesis. Upon activation, it recruits the coactivator PGC-1α to the promoters of its target genes. Remarkably, PGC-1α can be deacetylated by SIRT1, which enhances the transcriptional activity of PPARα, promotes FAO in liver, and contributes to protecting the liver from steatosis^6^. For example, in HFD fed mice with liver specific SIRT1 knockout, increased acetylation of PGC-1α impairs PPARα activation, resulting in decreased FAO, enhanced liver lipid accumulation, and increased production of pro-inflammatory cytokines^111^. Moreover, Jiang et al. found that alcohol intake suppresses SIRT1 expression and its nuclear translocation, leading to reduced PPARα activity and consequently downregulating the expression of carnitine palmitoyl transferase 1A (CPT-1A), a key enzyme involved in the regulation of FAO. Targeting SIRT1 with SGA can upregulate PPARα and downregulate the expression of lipogenic genes (e.g., SREBP-1c, ACC1, and FAS), thereby alleviating alcohol-induced liver lipid accumulation and steatosis^79^. In addition, PPARγ primarily regulates the generation, differentiation, and function of adipocytes^112^. Recent studies have demonstrated that Antrodan, a β-glucan extracted from Antrodia cinnamomea, activates the adenosine 5'-monophosphate (AMP)-activated protein kinase (AMPK) signaling pathway, increases the NAD⁺/NADH ratio, and induces the expression of SIRT1. Subsequently, SIRT1, in coordination with phosphorylated AMPK, suppresses the transcriptional activities of PPARγ and SREBP-1c, thereby reducing the expression of FAS and ACC, inhibiting liver lipogenesis, and promoting fatty acid oxidation^112^. Taken together, the PPAR signaling pathway is regulated by SIRT1 through multiple mechanisms and plays a crucial role in liver lipid biosynthesis.

### PI3K/Akt signaling pathway

The phosphoinositide 3-kinase/protein kinase B (PI3K/Akt) signaling pathway is a key regulator of cell proliferation, survival, metabolism, and migration^113^. Its activation relies on the interaction between ligands and receptor tyrosine kinases, which triggers PI3K to catalyze the conversion of phosphatidylinositol-4,5-bisphosphate (PIP2) into phosphatidylinositol-3,4,5-trisphosphate (PIP3). PIP3 subsequently recruits Akt to the plasma membrane, where it undergoes phosphorylation and activation, thereby modulating diverse cellular functions^114^, ^115^. Notably, dysregulation of PI3K/Akt signaling pathway has been implicated in various pathological conditions, including tumorigenesis, neurodegenerative diseases, and metabolic disorders^116^.

Currently, mounting research has indicated that the PI3K/Akt signaling pathway is closely associated with a range of chronic liver diseases^117^, ^118^. Zhang et al. demonstrated that SIRT1 suppresses PI3K activation, thereby reducing the phosphorylation of Akt. The decreased phosphorylation of Akt leads to a subsequent reduction in mammalian target of rapamycin (mTOR) phosphorylation. As a major negative regulator of autophagy, inactivation of mTOR alleviates its inhibitory effect on the transcription factor EB (TFEB), allowing TFEB to translocate into the nucleus and activate the expression of proteins involved in autophagy, including microtubule-associated protein light chain 3 (LC3) and FUN14 domain-containing 1 (FUNDC1). The resulting activation of autophagy facilitates the degradation of senescence-associated cellular components, including oxidized proteins and damaged organelles, thereby attenuating the senescence-associated secretory phenotype (SASP), suppressing hepatocyte senescence, and ameliorating MASLD^119^. Intriguingly, Koga et al. found that endoplasmic reticulum (ER) stress relieved the inhibition of glycogen synthase kinase-3β (GSK3β) by suppressing the PI3K/Akt signaling pathway. Activated GSK3β directly acted on the promoter region of SIRT1 and enhanced its transcription. Subsequently, pharmacological inhibition of SIRT1 with selisistat (EX-527, SIRT1 inhibitor) markedly attenuated hepatocyte death and ameliorated liver injury induced by tunicamycin-triggered ER stress in mice. Further investigation revealed that the ER stress-induced upregulation of SIRT1 may have aggravated hepatocyte apoptosis and inflammation by promoting the deacetylation of p53 and p65^120^. Collectively, SIRT1 may exert divergent roles in PI3K/Akt signaling under different pathological conditions of liver disease.

### Nrf2 signaling pathway

The nuclear factor erythroid 2-related factor 2 (Nrf2) signaling pathway represents a central cellular defense mechanism against oxidative stress and electrophilic toxicity^121^. Under physiological homeostasis, Nrf2 remains in a low-activity state through its interaction with the cytoplasmic protein Kelch-like ECH-associated protein 1 (Keap1), which promotes its ubiquitination and subsequent proteasomal degradation. Upon exposure to oxidative stress, electrophilic compounds, or inflammatory environments, cysteine residues on Keap1 are oxidatively modified, resulting in conformational changes that lead to the release of Nrf2. Then the released Nrf2 translocates into the nucleus, where it binds to antioxidant response elements (AREs) and initiates the transcription of downstream antioxidant genes^122^, ^123^. Therefore, activation of the Nrf2 signaling pathway can exert a protective effect against oxidative stress in various liver diseases through regulating the expression of antioxidant genes.

Recent studies have shown that the Nrf2 signaling pathway can be activated by SIRT1, thereby enhancing the antioxidant and anti-inflammatory effect of hepatocytes^7^. In a HFD-induced mouse model of MASLD, activation of the SIRT1/Nrf2 signaling pathway was shown to restore the activity of antioxidant enzymes (e.g., SOD), reduce liver ROS production, and subsequently improve lipid metabolic dysregulation and liver injury associated with MASLD^45^. In another study on MASLD, Yang et al. demonstrated that activation of the SIRT1/Nrf2 signaling pathway in PA-induced AML-12 cells inhibited ferroptosis and inflammation. However, this protective effect was markedly attenuated following SIRT1 inhibition by using EX-527, further confirming the involvement of SIRT1 in regulating the Nrf2 signaling pathway to protect against liver injury^124^. Moreover, Abu-Risha et al. demonstrated that SIRT1 directly activates the Nrf2 signaling pathway through its deacetylase activity, exerting antioxidant and anti-inflammatory effects in alcohol-induced liver fibrosis in rats, thereby ameliorating the progression of liver fibrosis^125^. In summary, SIRT1 modulates a series of cellular events by regulating Nrf2 activation, nuclear translocation, and downstream transcriptional activity, thereby orchestrating a series of cellular events that help maintain redox balance and mitigate excessive inflammation in the liver.

### AMPK signaling pathway

AMPK is a cellular energy sensor composed of α, β, and γ subunits that monitors changes in the AMP to adenosine triphosphate (ATP) ratio^126^. When intracellular ATP levels are reduced, leading to an increased AMP/ATP ratio, AMP or ADP can directly bind to the γ subunit. This binding promotes phosphorylation at threonine 172 (T172) on the α subunit, activating the AMPK signaling pathway and subsequently enhancing downstream energy-producing processes^127^. Of note, AMPK activation confers protection in the progression of liver diseases by modulating lipid metabolism, enhancing insulin sensitivity, and suppressing inflammatory responses. Conversely, AMPK dysfunction may exacerbate liver steatosis, accelerate fibrosis, and even contribute to the development of HCC^128^, ^129^.

Numerous investigations have demonstrated that SIRT1 is involved in the regulation of the AMPK signaling pathway and plays a critical role in suppressing liver inflammatory responses. An interesting experiment by Zou et al. has shown that swimming exercise can activate the SIRT1/AMPK signaling pathway in the liver of zebrafish models of MASLD, which on one hand, suppressed the expression of downstream lipogenic genes, and on the other hand, promoted the expression of genes involved in FAO. This dual regulation contributed to the reduction of liver TG and TC levels, thereby alleviating lipid accumulation in the liver. Meanwhile, activation of the AMPK signaling pathway also attenuates liver inflammation, alleviates oxidative stress, and suppresses hepatocyte apoptosis, thereby improving HFD-induced MASLD in zebrafish^130^. Notably, the AMPK signaling pathway can be activated by certain polyphenols through the modulation of SIRT1. For example, kaempferol, a flavonoid polyphenolic compound, was able to significantly attenuate MASLD symptoms in type 2 diabetic mice by activating the SIRT1/AMPK signaling pathway^131^. Besides, apple polyphenol extract reduces lipid deposition in free fatty acid (FFA)-treated HepG2 cells by indirectly activating AMPK through SIRT1 via deacetylation and activation of liver kinase B1 (LKB1)^132^. Similarly, resveratrol delays organic aging and attenuates oxidative damage in the liver by indirectly activating AMPK through SIRT1-mediated deacetylation and activation of LKB1^133^. In addition, vitamin D has been found to promote the SIRT1/AMPK signaling pathway in AML-12 cells, which reduces liver fat accumulation and alleviates liver inflammation^134^. Moreover, the SIRT1/AMPK signaling pathway can be stimulated by certain bacilli, such as Lactobacillus plantarum 69-2, which can regulate the intestinal flora and its metabolites through the liver-intestinal axis, thereby activating the SIRT1/AMPK signaling pathway in the liver, restoring the antioxidant activity of the liver, and effectively alleviating liver diseases^135^. Overall, SIRT1 facilitates the activation of the AMPK signaling pathway, which in turn modulates liver energy metabolism, suppresses inflammatory responses, and ultimately delays the progression of liver diseases.

## Clinical utility of SIRT1 in liver disease

Accumulating clinical evidence highlights the crucial role of SIRT1 in liver resistance and metabolic balance. Currently, several pharmacological modulators of SIRT1 have demonstrated potential for application in the treatment of liver diseases in clinical studies (**Table 1**). For example, resveratrol, a known activator of SIRT1, has shown beneficial effects in clinical studies involving MASLD patients. In a randomized, placebo-controlled, double-blind trial of 50 MASLD patients, daily administration of 500 mg resveratrol for three consecutive months led to significant improvements compared with the placebo group. These included reductions in anthropometric parameters (body weight, body mass index, and waist circumference), serum AST/ALT levels, inflammatory cytokines (such as TNF-α and IL-6), and hepatic steatosis^136^. Another randomized controlled trial in 60 MASLD patients also reported that twice-daily administration of 150 mg resveratrol for three months exerted a notable liver protective effect and improved insulin resistance. Unfortunately, this study did not evaluate SIRT1 expression or its downstream targets, leaving uncertainty as to whether the observed effects were mediated via SIRT1^137^. Intriguingly, Heebøll et al. administered high-dose resveratrol (1.5 g/day for 6 months) to 28 patients with MASLD, resulting in a 3.8% reduction in liver lipid content. However, histological improvement was not observed, and there were no significant changes in anthropometric parameters or the expression of genes related to SIRT1^138^. By contrast, a large-scale clinical study by Ma et al. in 472 elderly patients with type 2 diabetes demonstrated that daily supplementation with 500 mg resveratrol significantly enhanced SIRT1 activity, reduced serum triglyceride levels, and improved glucose metabolism^139^. The aforementioned studies suggest that escalating the dose of resveratrol does not necessarily lead to better histological results. In addition, resveratrol has multiple molecular targets. Beyond activating SIRT1, it can also target Keap1, NLRP3, and cyclooxygenase-2 (COX-2), activating the Nrf2 and inhibiting NF-κB signaling pathway^140^. Precisely due to its multi-targeted mechanism, the efficacy of resveratrol varies across patients and pathological conditions. In patients with coexisting underlying metabolic disorders, resveratrol may exert beneficial effects by improving insulin resistance and attenuating inflammation. Whereas in patients with isolated steatotic liver disease, its impact on liver lipid accumulation appears relatively limited^141^. Accordingly, while multiple biological effects of resveratrol may offer potential advantages, they also contribute to the complexity of clinical research. Moreover, resveratrol exhibits low intestinal bioavailability, with the majority of unmetabolized compound interacting with the gut microbiota and subsequently being converted into more absorbable derivatives^142^. Therefore, interindividual differences in gut microbiota composition and abundance may lead to substantial variability in the effective absorption of resveratrol at equivalent doses. Overall, the multi-targeted effects of resveratrol, along with variations in patient conditions and individual differences included in clinical studies, may account for the discrepancies observed in clinical results.

Other natural compounds, particularly phenolic compounds, have not yet been evaluated in clinical studies, but preclinical investigations have demonstrated their considerable potential in targeting SIRT1 for the treatment of liver diseases^143^. For example, the polyphenolic compound rosmarinic acid, which is extracted from plants such as rosemary and perilla, has been shown to upregulate SIRT1 expression and suppress liver inflammation^105^. Besides, flavonoids like kaempferol and dihydromyricetin further highlight the therapeutic potential of natural compounds^144^, ^145^. They can reduce ALT, AST, ROS, and pro-inflammatory cytokines (e.g., IL-1β, TNF-α), with elevated SIRT1 expression^131^. Similarly, dihydromyricetin has been shown to activate SIRT1 protein, thereby exerting liver protective effects through reducing ALT, AST, IL-1β, IL-6, and TNF-α levels^80^. Moreover, saponins such as ginsenoside and saikosaponin A exhibit notable benefits through SIRT1 activation. Ginsenoside, upon activating SIRT1, can decrease ALT, AST, superoxide dismutase (SOD), and glutathione S-transferase (GST) levels^107^. Saikosaponin A improves liver function by lowering ALT, AST, TC, and TG through activating SIRT1^79^.

Beyond natural compounds, conventional drugs also exhibit SIRT1-modulating properties. Metformin, a widely used biguanide, has been shown to decrease ALT, AST, TC, and TG levels, as well as increase SIRT1 expression. In 2018, Chalasani et al. administered a leucine-metformin-sildenafil combination (NS-0200) to 91 patients with MASLD for 16 weeks. The triple combination drug NS-0200 exerted synergistic activation effects by centrally targeting SIRT1, aiming to alleviate MASLD. As a result, although the full cohort did not show a significant reduction in liver fat content compared to placebo, a post-hoc analysis revealed that high-dose NS-0200 (1.1 g leucine/0.5 g metformin/1.0 mg sildenafil twice daily) resulted in a 15.7% relative reduction in liver fat in a subgroup of 35 patients with elevated ALT levels^146^. Collectively, these findings highlighted the diverse array of compounds that can target SIRT1, paving the way for the development of innovative strategies against liver diseases.

## Future prospects

As research progresses, the role of SIRT1 in regulating physiological processes in liver diseases has become increasingly evident. Recent research has focused on exploring the function and molecular mechanisms of SIRT1 in liver diseases, aiming to comprehensively understand its roles in liver pathophysiology. In these studies, the precise regulation of SIRT1 expression is essential. However, traditional gene knockout mice and cell lines are typically generated via homologous recombination, a method that is both inefficient and time-consuming. Notably, the advent of genome‑editing technologies, particularly the clustered regularly interspaced short palindromic repeats (CRISPR) system, has dramatically improved the efficiency of generating gene knockout animal and cell models^147^, ^148^. One illustrative example is the CRISPR/Cas9 system, a versatile genome-editing tool that has been widely applied across various research and clinical domains. It employs a synthetic single‑guide RNA to direct the Cas9 endonuclease to specific DNA loci, thereby enabling precise and targeted genetic modifications^149^. Currently, the CRISPR/Cas9 system has been employed to knock out the SIRT1 gene. For example, Jeon et al. established a SIRT1 knockout model in B16F1 cells (a murine melanoma cell line) using the CRISPR/Cas9 system, and observed a significant reduction of melanin content in the SIRT1-deficient cells^150^. In addition to constructing preclinical models, novel CRISPR technologies, including CRISPR activation (CRISPRa) and CRISPR interference (CRISPRi), enable the modulation of gene expression without inducing permanent alterations to the DNA sequence, thereby exhibiting great potential for therapeutic applications in gene therapy^151^, ^152^. In future research, exploring the precise upregulation of SIRT1 through CRISPRa to ameliorate certain liver diseases, such as MASLD, may represent a feasible and promising strategy. However, the clinical translation of CRISPR still requires further research to improve its specificity, minimize off-target effects, and overcome challenges related to the immunogenicity of CRISPR components, as well as ethical and safety concerns. Overall, the CRISPR system represents a powerful approach for generating SIRT1 gene‑edited models, as well as a potential therapeutic strategy for targeting SIRT1 in the treatment of liver diseases.

Notably, SIRT1 is broadly expressed across multiple tissues, and systemic administration of SIRT1 activators may result in off-target effects, potentially disrupting lipid metabolism and other physiological functions in organs beyond the liver. Furthermore, the pleiotropic roles of SIRT1, particularly its immunomodulatory properties, can lead to divergent outcomes depending on the type and stage of liver disease. For example, SIRT1 has been shown to suppress pro-inflammatory polarization of macrophages during inflammatory responses in acute liver injury, whereas its activation may paradoxically exacerbate liver inflammation in murine models of hepatitis B^153^. Additionally, pharmacological SIRT1 activators like SRT2104 have demonstrated side effects in clinical trials, including headaches, gastrointestinal disturbances, dizziness, and nasopharyngitis, often mild but highlighting tolerability concerns. Other SIRT1 activators, such as resveratrol, exhibit low bioavailability and can interact with multiple targets beyond SIRT1. Therefore, the precise delivery of SIRT1 modulating agents to the liver is also essential for its functional studies and clinical translation. In recent years, nanomedicine, an interdisciplinary frontier harnessing nanoscale innovation for diagnostic and therapeutic applications, has emerged as a transformative paradigm in modern medicine. This advancement is driven by the capacity of engineered nanomaterials to achieve targeted and biocompatible drug delivery with high precision^154^, ^155^. Nano-based drug delivery systems enable cell-specific therapeutic cargo delivery, revolutionizing the detection and management of diverse pathologies. Recently, Thakur et al. developed a nanoparticle-based delivery system encapsulating Honokiol, which can activate SIRT3 (another member of the mitochondrial SIRT family), and conferred targeted protection to zebrafish lateral line neuromast hair cells against cisplatin-induced injury^156^. Similarly, given the extensive research on liver-targeted nano-based drug delivery systems, encapsulating SIRT1 modulators into specially designed liver-targeted functionalized nanocarriers represents a promising strategy for the treatment of liver diseases.

## Conclusion

In conclusion, SIRT1 occupies a crucial role in liver physiology and pathology, with its distinct functions in MASLD, ALD, HCC and liver fibrosis now increasingly well elucidated. A growing repertoire of pharmacological modulators of SIRT1 has demonstrated efficacy in preclinical models, although their clinical translation critically depends on delivery systems that combine safety with high precision and efficiency. Achieving this requires precise optimization of lipid composition, surface functionalization and particle size to control biodistribution, enhance target specificity and minimize off‑target effects. Ultimately, the convergence of mechanistic insights, advanced delivery technologies, and clinical rigor will be essential for the development of next-generation SIRT1-targeted therapies in liver diseases.

## Figures and Tables

**Table 1:** Potential SIRT1-targeted agents for treatment of liver disease

Categories	Names	Modulators	Chemical structures	Effects	References
Saponin	Ginsenoside	SIRT1 agonist	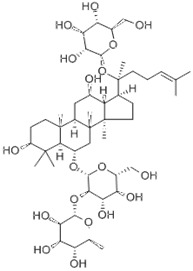	ALT, AST↓ SOD, GST↓	107
	Saikosaponin A	SIRT1 agonist	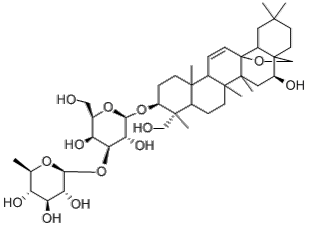	ALT, AST↓ TC, TG↓	79
Phenols	Resveratrol	SIRT1 agonist	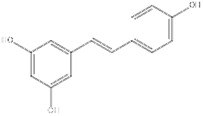	IL-1β, IL-6↓ TNF-α↓ TG↓	136,137,138, 139
	Rosmarinic acid	SIRT1 agonist	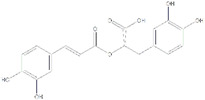	SREBP1↓ IL-6↓ TNF-α↓ P53 ↓	105
Flavonoid	Kaempferol	SIRT1 agonist	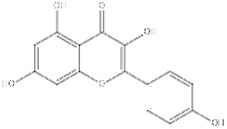	ALT, AST↓ ROS↓ IL-1β↓ TNF-α↓	131
	Dihydromyricetin	SIRT1 agonist	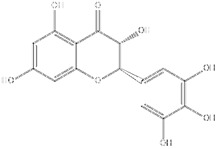	ALT, AST↓ IL-1β, IL-6↓ TNF-α↓	80
				
Biguanide	Metformin	SIRT1 agonist	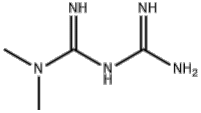	ALT, AST↓ TC, TG↓	146

## Abbreviations

ACC: acetyl coenzyme A carboxylase
ADH: alcohol dehydrogenase
ALD: alcohol-associated liver disease
ALT: glutamic pyruvic transaminase
AMPK: adenosine 5'-monophosphate (AMP)-activated protein kinase
ANIT: alpha-naphthylisothiocyanate
AREs: antioxidant response elements
AST: aspartate transaminase
ATP: adenosine triphosphate
BAFF: B-cell activating factor
Bcl-2: B-cell lymphoma 2
BDL: bile duct ligation
CAT: catalase
CCl_4_: carbon tetrachloride
CD: cluster of differentiation
CDK: cyclin-dependent kinase
COL1A1: Collagen type I
COX-2: cyclooxygenase 2
CPT-1A: carnitine palmitoyl transferase 1A
CRISPR: clustered regularly interspaced short palindromic repeats
CRISPRa: CRISPR activation
CRISPRi: CRISPR interference
Cy-3-G: cyanidin-3-O-β-glucoside
DAAs: direct-acting antiviral agents
DALYs: disability-adjusted life years
DNA: deoxyribonucleic acid
DMY: dihydromyricetin
E2F1: E2F transcription factor 1
ECM: extracellular matrix
EMT: epithelial-to-mesenchymal transition
FAO: fatty acid β-oxidation
FFA: free fatty acid
FOXO: forkhead box O
FUNDC1: FUN14 domain-containing 1
GCDCA: glycochenodeoxycholic acid
GDF15: growth differentiation factor 15
GPX: glutathione peroxidase
GST: glutathione S-transferase
HCV: hepatitis C virus
HCC: hepatocellular carcinoma
HFD: high-fat diet
HIC1: hypermethylated in cancer 1
HO-1: heme oxygenase-1
HPCs: hepatic progenitor cell
HSC: hepatic stellate cell
HSEC: hepatic sinusoidal endothelial cell
IKKα: inhibitor of κB kinase α
IL-1β: interleukin 1β
IL-6: interleukin 6
IRS-2: insulin receptor substrate 2
Keap1: Kelch-like ECH-associated protein 1
LC3: microtubule-associated protein light chain 3
LKB1: liver kinase B1
LPS: lipopolysaccharide
MASLD: metabolic dysfunction-associated steatotic liver disease
MCD: methionine and choline deficient
MCP-1: monocyte chemoattractant protein-1
miRNAs: microRNAs
MEOS: microsomal ethanol oxidase
mTOR: mammalian target of rapamycin
NAD^+^: nicotinamide adenine dinucleotide
NAMPT: nicotinamide phosphoribosyl transferase
NASH: non-alcoholic steatohepatitis
ncRNAs: non-coding RNAs
NIAAA: National Institute on Alcohol Abuse and Alcoholism
NLRP3: NOD-like receptor protein 3
NLS: nuclear localization signal
NESs: nuclear export sequences
NQO1: NAD(P)H quinone dehydrogenase 1
NF-κB: nuclear factor kappa-B
Nrf2: nuclear factor erythroid 2-related factor 2
PA: palmitic acid
PAMPs: pathogen-associated molecular patterns
PET/CT: positron emission tomography/computed tomography
PGC-1α: Peroxisome proliferator-activated receptor γ coactivator 1α
PI3K/Akt: phosphoinositide 3-kinase/protein kinase B
PIP2: phosphatidylinositol-4,5-bisphosphate
PIP3: phosphatidylinositol-3,4,5-trisphosphate
PPAR: peroxisome proliferator-activated receptor
PPREs: peroxisome proliferator response elements
ROS: reactive oxygen species
SalA: Salvianolic acid A
SASP: senescence-associated secretory phenotype
SCFAs: short chain fatty acids
SGA: Saikogenin A
SIRT1: silent information regulator 1
SOD: superoxide dismutase
SREBP-1: sterol regulatory element-binding protein 1
SSa: Saikosaponin A
TAA: thioacetamide
TC: total cholesterol
TFEB: transcription factor EB
TG: triglyceride
TGF-β: transforming growth factor-β
TNF-α: tumor necrosis factor
3'UTR: 3' untranslated region
α-SMA: α-smooth muscle actin
